# Microbial interactions and the ecology and evolution of Hawaiian Drosophilidae

**DOI:** 10.3389/fmicb.2014.00616

**Published:** 2014-12-18

**Authors:** Timothy K. O’Connor, Parris T. Humphrey, Richard T. Lapoint, Noah K. Whiteman, Patrick M. O’Grady

**Affiliations:** ^1^Ecology and Evolutionary Biology, University of ArizonaTucson, AZ, USA; ^2^Environmental Science, Policy and Management, University of California BerkeleyBerkeley, CA, USA

**Keywords:** Hawaiian *Drosophila*, *Scaptomyza*, symbiosis, fungi, *Pseudomonas*, herbivory, adaptive radiation

## Abstract

Adaptive radiations are characterized by an increased rate of speciation and expanded range of habitats and ecological niches exploited by those species. The Hawaiian Drosophilidae is a classic adaptive radiation; a single ancestral species colonized Hawaii approximately 25 million years ago and gave rise to two monophyletic lineages, the Hawaiian *Drosophila* and the genus *Scaptomyza*. The Hawaiian *Drosophila* are largely saprophagous and rely on approximately 40 endemic plant families and their associated microbes to complete development. *Scaptomyza* are even more diverse in host breadth. While many species of *Scaptomyza* utilize decomposing plant substrates, some species have evolved to become herbivores, parasites on spider egg masses, and exploit microbes on living plant tissue. Understanding the origin of the ecological diversity encompassed by these nearly 700 described species has been a challenge. The central role of microbes in drosophilid ecology suggests bacterial and fungal associates may have played a role in the diversification of the Hawaiian Drosophilidae. Here we synthesize recent ecological and microbial community data from the Hawaiian Drosophilidae to examine the forces that may have led to this adaptive radiation. We propose that the evolutionary success of the Hawaiian Drosophilidae is due to a combination of factors, including adaptation to novel ecological niches facilitated by microbes.

## INTRODUCTION

Symbioses are broadly defined as persistent interactions between two or more species. While one view of symbioses is restricted to mutualistic relationships, most biologists now consider any type of long-standing interaction between species (e.g., commensalism, mutualism, parasitism) as a symbiotic relationship (Bradford and Schwab, 2013). Many insects have developed intimate evolutionary interactions with microbes that enhance nutrient acquisition or reproduction (Moran and Baumann, 2000; Currie et al., 2003, 2006; Mikheyev et al., 2007; Werren et al., 2008; Hansen and Moran, 2014), or defense against natural enemies (Oliver et al., 2009; Jaenike et al., 2010). A different type of relationship is seen in saprophagous insects, such as many fly species in the family Drosophilidae, which require microbes to break down plant material and make nutrients available for uptake.

Yeasts and bacteria associated with drosophilid flies can influence mating behavior, oviposition behavior, larval feeding choice, and food processing, and these ecological roles can have important evolutionary consequences for insects. Here we propose the Hawaiian Drosophilidae as a model system for studying the role of microbial associations in insect diversification. We focus on two systems: (1) the fungal associates of the largely saprophagous Hawaiian *Drosophila* lineage and (2) the bacterial species encountered by drosophilids, especially herbivorous members of the genus *Scaptomyza*.

## HAWAIIAN DROSOPHILIDAE

The Hawaiian Drosophilidae is one of the best-characterized examples of an adaptive radiation (Carson and Kaneshiro, 1976). This group includes 687 described species (Magnacca and Price, 2012) and 200–300 more taxa that await description (O’Grady et al., 2011). Hawaiian Drosophilidae have adapted to a diverse array of niches and plant substrates (Kambysellis and Craddock, 1997), and their interactions with microbes are a central part of *Drosophila* ecology. Microbes have been implicated in providing direct and indirect nutrition sources (Northrop, 1918; Starmer and Aberdeen, 1990), generating chemosensory signals (Dobzhansky et al., 1956; Melcher and Pankratz, 2005), and extensively colonizing larvae and adults (Gilbert, 1980; Ganter, 1988; Coluccio et al., 2008). Although microbes can influence insect ecology (Feldhaar, 2011), promote speciation (Brucker and Bordenstein, 2012, 2013; Joy, 2013), and promote niche differentiation (Janson et al., 2008; Joy, 2013), the potential role of microbes in the diversification of Hawaiian Drosophilidae has not been explored in depth.

Drosophilidae is the oldest known lineage of endemic Hawaiian plants or insects (Price and Clague, 2002). A single colonizing species is estimated to have arrived in the Hawaiian Islands ∼25 million years ago (Thomas and Hunt, 1993; Russo et al., 1995), although recent estimates suggest a slightly older age (Tamura et al., 2004; Obbard et al., 2012). The Hawaiian Drosophilidae has since radiated into two species-rich lineages, the endemic Hawaiian *Drosophila* and the cosmopolitan genus *Scaptomyza*. Although the inclusion of *Scaptomyza* within a larger *Drosophila* group is confusing taxonomically, this is due to the large-scale polyphyly of the genus *Drosophila* (O’Grady and DeSalle, 2008; O’Grady et al., 2008a,b; O’Grady and Markow, 2009; O’Grady, 2010). The Hawaiian Drosophilidae (Hawaiian *Drosophila*+ *Scaptomyza*) is strongly supported as monophyletic in every rigorous phylogenetic study (Throckmorton, 1975; Thomas and Hunt, 1991, 1993; Baker and DeSalle, 1997; Remsen and DeSalle, 1998; Bonacum, 2001; Remsen and O’Grady, 2002; O’Grady and DeSalle, 2008; O’Grady et al., 2011). Most members of the genus *Drosophila*, including those endemic to Hawaii, are saprophagous and have adapted to a diverse array of substrates for oviposition, larval development and adult nutrition (Markow and O’Grady, 2005, 2008). While many *Scaptomyza* species are saprophagous on a variety of larval substrates, including plant leaves and flowers, some now specialize on spider egg sacs or land snails (Magnacca et al., 2008), and herbivory has evolved at least once within this lineage (Lapoint et al., 2013).

## HAWAIIAN *Drosophila* AND ASSOCIATED YEASTS

**FIGURE 1:**
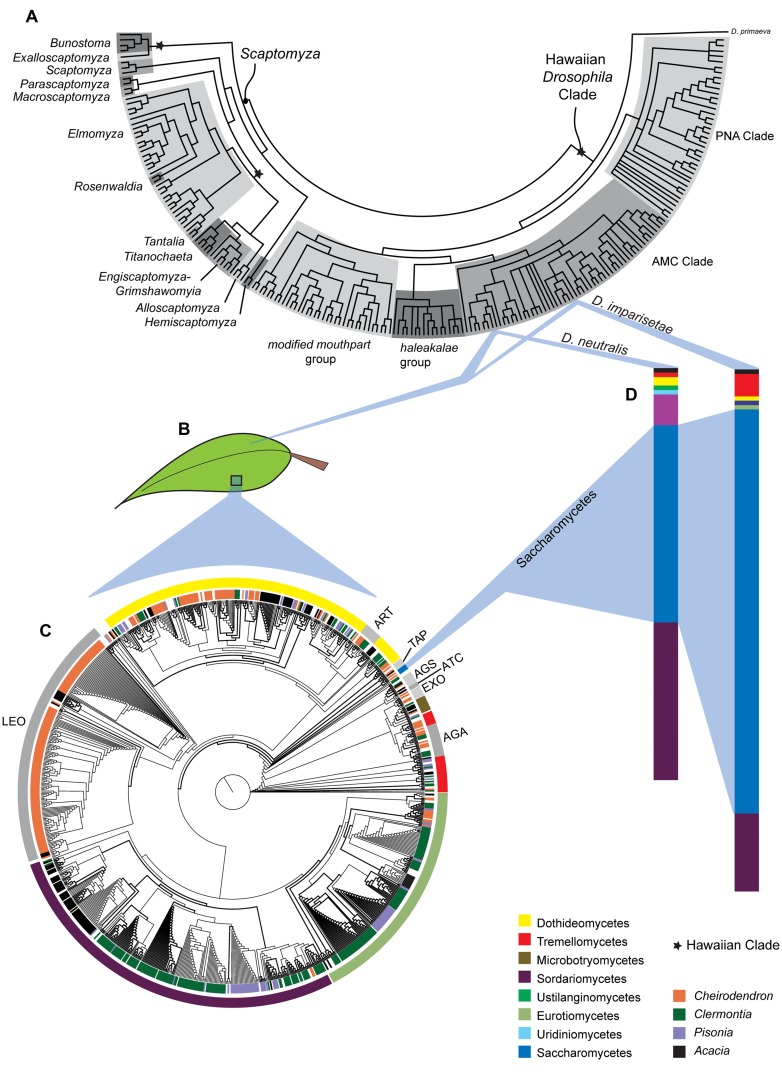
**Ecological and evolutionary relationships between Hawaiian Drosophilidae, their endemic host plants and fungal decomposers. (A)** Phylogenetic relationships among the Hawaiian *Drosophila* and *Scaptomyza*. **(B)** Two species in the AMC Clade, *Drosophila imparisetae* and *Drosophila neutralis*, both oviposit in rotting leaves of Araliaceae (*Cheirodendron*). **(C)** The phylogeny of the fungal taxa present in decomposing leaves from *Cheirodendron* (and other Hawaiian plant taxa) shows a diverse fungal community. The inner circle indicates the plant of origin, while the outer circle indicates fungal taxonomy. **(D)** The relative dearth of Saccharomycetes representatives in leaf communities is interesting given the prevalence, this fungal class in and on the bodies of the adult *Drosophila* species sampled. Aspects of this figure have been modified with permission from O’Grady et al. (2011),Ort et al. (2012), and Lapoint et al. (2013).

The Hawaiian Drosophilidae (**Figure 1A**) utilize nearly 40% of the native Hawaiian plant families and an array of substrate types (leaves, bark, fruits, sap flux, fungus; Magnacca et al., 2008). Hawaiian *Drosophila* adults use volatile compounds as cues to identify host plants and stimulate mating and oviposition, although the identity and origin of these cues are unknown (Ohta, 1978). Among the closely related species in the cactophilic *Drosophila repleta* group, such cues can include byproducts of microbial metabolism (Fogleman and Foster, 1989), raising the possibility that host finding in the Hawaiian Drosophilidae may also be microbially mediated.

Ort et al. (2012) surveyed four endemic Hawaiian plants (**Figure 1B**) to determine whether microbial communities played a role in host plant specificity in Hawaiian *Drosophila*. Over 160 OTUs, representing 113 genera and 13 fungal classes, were discovered (**Figure 1C**). Ort et al. (2012) found little sharing of fungal taxa between different substrates, and fungal communities differed significantly between substrate type (e.g., leaves *vs*. stems) and among plant genera. It is clear that different substrates support correspondingly distinct fungal communities, which may provide unique oviposition cues or nutrition to flies that use those substrates.

Relative to their host plants, the fungal communities of the two *Drosophila* species examined, *Drosophila imparisetae* and *Drosophila neutralis*, were relatively simple: only seven or eight fungal lineages were present (**Figure 1D**). This suggests that *Drosophila* vector a limited number of fungal species from plant to plant. Interestingly, the most abundant fungal class associated with *Drosophila* adults, Saccharomycetes, was only modestly represented in the *Cheirodendron* leaf samples (**Figure 1C**), suggesting that Hawaiian flies select and vector their own yeasts from rotting plant to rotting plant, as in the cactophilic *Drosophila* (Barker and Starmer, 1982).

*Drosophila*-associated microbes may contribute to reproductive isolation of closely related species, which is critical to sustaining adaptive radiations. In the cactophilic species *Drosophila buzzatii*, heritable variation in oviposition behavior is mediated by attraction to different yeasts (Barker et al., 1994), which might contribute to assortative mating among genotypes. Isolation among races and species of cactophilic *Drosophila* species may have evolved due to chemical variation in larval substrates that are a combination of necrotic host plant tissues and microbial communities (Etges and de Oliveira, 2014). Combined with evidence that bacterial communities play a direct role in mating preference in *Drosophila melanogaster* (Sharon et al., 2010), this suggests that microbes can directly or indirectly influence speciation of drosophilid flies through mechanisms that are dependent on larval feeding substrates. Ohta (1980) provides strong evidence that post-mating barriers involving large chromosomal inversion in Hawaiian *Drosophila* can be explained by variation in host plant use. Thus, the combined effects of host plant phenotype and microbial community phenotypes have likely played an important role in driving the diversification of Hawaiian Drosophilidae.

## *Scaptomyza* AND ASSOCIATED BACTERIA

Most drosophilid flies likely feed on microbes associated with rotting vegetation or on the fruiting bodies of fungi. However, in a few lineages (including *Scaptomyza*), feeding on living plant tissues as a primary source of nutrition (herbivory) has evolved. This transition to herbivory is a remarkable feature of the Hawaiian Drosophilidae radiation, the challenges of which are underscored by the paucity of insect orders with herbivorous members (Mitter et al., 1988). Given the change in the nature of the relationship between *Scaptomyza* and its food source, the nature of its relationship to its microbial communities is expected to also change. It is likely that herbivorous drosophilid lineages encounter distinct groups of plant-associated microbes that may influence fly adaptation to these larval substrates.

**FIGURE 2:**
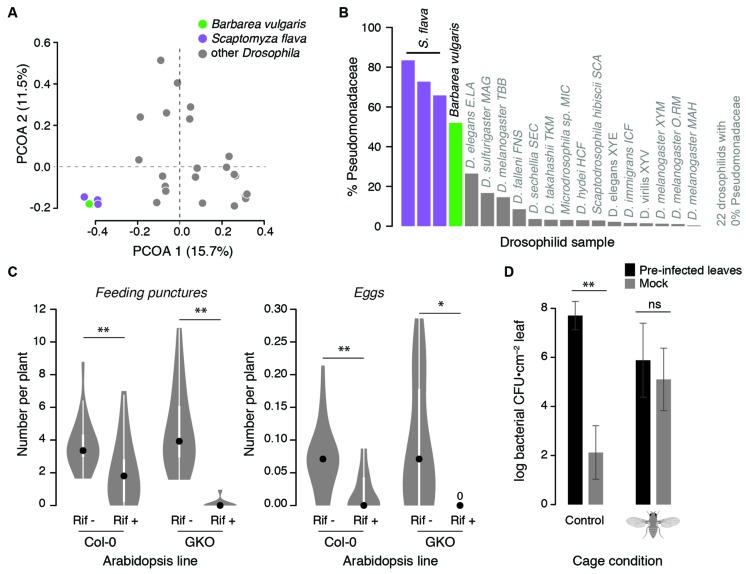
**Observational and experimental evidence linking *Scaptomyza* ecology with microbes. (A,B)** The gut bacterial community of *Scaptomyza flava* larvae more closely resembles that of a host plant (*Barbarea vulgaris*) than that of other drosophilids. Field-collected *Scaptomyza flava* and *Barbarea vulgaris* microbial communities were characterized with Illumina sequencing (according to Caporaso et al., 2012) and compared to other *Drosophila* communities sequenced by Chandler et al. (2011). **(A)** Principal coordinate analysis (PCOA) of unweighted UniFrac distances summarizing differences in gut bacterial community composition among species. Gut bacteria of *Scaptomyza flava* are significantly different from those of other drosophilids (PERMANOVA, *P* < 0.001). **(B)** The relative abundance of bacteria classified to Pseudomonadaceae is greater in *Scaptomyza flava* than other drosophilids and comparable to levels found in *Barbarea vulgaris*. **(C)** Pre-treating *Scaptomyza flava* with antibiotics reduces feeding and fecundity on *Arabidopsis thaliana.* Lab-reared flies were fed for 4 days on 5% sucrose with (Rif+) or without (Rif–) 50 μg/mL rifampicin (Sigma). Treatments were delivered into feeding chambers with 5 μL microcapillary tubes that were refreshed daily. After 4 days, all surviving flies were randomized to cages with plants of either line (wild-type Col-0 or glucosinolate knock-out GKO) and were allowed to feed and oviposit for 24 h, after which feeding puncture and eggs on each plant were counted. The number of feeding punctures and eggs per plant were normalized by number of flies released into each cage (each Rif– condition had 14 females; each Rif+ condition had 23 females). Data are presented as boxplots with 50% quantiles around the medians (dots) and symmetrical marginal frequency distributions. **P < 0.01, *P < 0.05 (Mann–Whitney *U*-test, two-tailed). **(D)** *Scaptomyza flava* females enhance transmission of *Pseudomonas syringae* between *A. thaliana* leaves in the laboratory; *Pseudomonas syringae* grew in un-treated leaves only when flies were present. Three lower leaves of 5 weeks old *A. thaliana* Col-0 were infected with 10^5^/ml *Pseudomonas syrinage* pv. maculicola str. 4326. Four days later, leaves were removed and petioles were inserted into 60 mm petri dishes containing 1% Phytagel to maintain leaf hydration. Equal numbers of infected or un-infected leaves were randomized into one of two mesh cages. Into one of these cages, we released 20 adult female *Scaptomyza flava* for 2 days after which feeding punctures were counted on all leaves. Leaf discs were taken and homogenized in 10 mM MgSO_4_ and dilution-plated onto King’s B medium, and fluorescent colonies were counted 4 days later. Error bars indicate standard errors; **P < 0.01, ‘ns’ non-significant, unpaired *t*-tests on log_10_-transformed CFU counts.

Leaf-mining *Scaptomyza* larvae are extensively colonized by plant-associated bacteria during feeding. Gut bacterial composition of larval *Scaptomyza flava* collected from leaves of wild *Barbarea vulgaris* (Brassicaceae) resembled that of their host plant more than any other drosophilid: 99.7% of gut bacterial sequences matched OTUs found in *Barbarea vulgaris* (**Figure 2A**). Pseudomonadaceae predominated in *Scaptomyza flava* guts and *Barbarea vulgaris* leaves (**Figure 2B**). Also found in both samples were *Enterobacter cloacae*, which includes some strains that degrade isothiocyanates (Tang et al., 1972), a group of potent foliar toxins in the Brassicaceae that are released upon plant wounding. Although untested, *Enterobacter cloacae* or other bacteria may supplement the endogenous isothiocyanate detoxification abilities of *Scaptomyza*, which involve modification of ancient evolutionarily conserved detoxification pathways (Gloss et al., 2014).

One way herbivorous *Scaptomyza* may have adapted to feeding on living plant tissue is by acquiring novel microbial symbionts that aid in substrate utilization, for instance by catabolizing polysaccharides or plant secondary compounds. Bacteria that can metabolize plant-derived molecules in ways that contribute to insect fitness are most likely to be found already associated with the novel substrate (reviewed in Janson et al., 2008; Mason et al., 2014). Indeed, recent work suggests *Scaptomyza flava* depends upon their gut bacteria for fitness within plants. Laboratory-reared *Scaptomyza flava* treated with the antibiotic rifampicin showed reduced feeding rates and lower fecundity on *Arabidopsis thaliana* (*Arabidopsis*) compared to control flies (**Figure 2C**). This effect does not appear to be due to direct antibiotic toxicity, because treatment and control flies survived at similar rates, nor interactions with plant defensive compounds. This is because results were consistent when flies were reared on wild-type *Arabidopsis* as well as a mutant line deficient in the production of two defensive compounds. Although particular bacteria have not been implicated, this experiment indicates that gut bacterial communities may be important for degrading or processing plant tissues.

Diffuse interactions between insects, bacteria, and plants may also be involved in the evolution and maintenance of herbivory. For instance, insects can vector plant-associated bacteria between plants, including common pathogens, to make those plants more suitable hosts. *Drosophila melanogaster* can serve as both host and vector to the plant pathogen *Erwinia carotovora* (Basset et al., 2000, 2003), and several insects vector *Pseudomonas syringae* between plants (Snyder et al., 1998; Stavrinides et al., 2009).

*Scaptomyza flava* adult female flies can enhance the transmission of a model pathogenic strain of *Pseudomonas syringae* (**Figure 2D**); *Pseudomonas syringae* moved from pre-inoculated *Arabidopsis* leaves to un-treated control leaves in cages with adult female flies added, while control leaves only showed background levels of non-*Pseudomonas syringae* bacteria in cages without flies (**Figure 2D**). Not only are *Pseudomonas* spp. widespread within tissues of *Scaptomyza flava* and its host plant, but *Pseudomonas syringae* are overrepresented within plant leaves damaged by a close relative, *Scaptomyza nigrita,* in the wild (Humphrey et al., 2014). Furthermore, experimental plant infection with *Pseudomonas* species can enhance feeding by adults of the specialist *Scaptomyza nigrita* in the laboratory, indicating that plant exposure to certain *Pseudomonas* spp. can induce susceptibility to this herbivore (Humphrey et al., 2014). Thus, the potential exists for drosophilid herbivores to frequently encounter and transmit *Pseudomonas* species between host plants in ways that enhance insect fitness. Frequent exposure to, and transmission of, defense-altering microbes can ultimately lead to novel and potentially mutualistic interactions between microbes and insects (Luan et al., 2012).

Inoculating plants with bacteria may allow *Scaptomyza* species to subvert anti-herbivore defense by exploiting mutual antagonism between plant defense pathways, including the canonical plant defense hormones salicylic acid (SA) and jasmonic acid (JA). The SA pathway, typically triggered by bacterial infections, represses the JA pathway, which is typically triggered by chewing herbivores (Thaler et al., 2012). Bacterial infection can thus alter plant chemistry in ways that also affect herbivores, including via mechanisms independent of SA–JA antagonism (Cui et al., 2002, 2005; Groen et al., 2013). The Colorado potato beetle was recently shown to locally disable plant defenses by secreting bacteria—including *Pseudomonas syringae*—into plant tissues while feeding (Chung et al., 2013), likely via SA–JA antagonism. Actively suppressing host plant defenses with bacteria may be a common behavior among herbivorous insects, including *Scaptomyza*.

Plant interactions with leaf-colonizing bacteria are ubiquitous, and the phenotypic impacts of bacteria on plants likely have always been a part of the context in which insect herbivory evolves. Indirect interactions are numerous in diverse ecological communities, and can impact selection on focal herbivore traits such as feeding preference both within (Utsumi et al., 2012) or between plant host individuals (Tack et al., 2012). Specifically, herbivores within the Hawaiian Drosophilidae hold great potential to shed light on the role of plant-mediated indirect effects in the ecology and evolution of herbivore traits given that the focal players are themselves—or are related to—genetic model organisms (Whiteman et al., 2011).

## HOST SHIFTS AND SYMBIOSES

Plant defenses, especially secondary compounds, are key obstacles to host plant switching for herbivorous insects (Ehrlich and Raven, 1964). The tendency of herbivorous insect lineages to specialize upon plants with similar secondary chemistry suggests that mechanisms to overcome these defenses may be difficult for already-specialized insects to evolve (Berenbaum et al., 1989). However, many microbes are known to degrade plant secondary compounds and structurally similar chemicals (Winkelmann, 1992). Freeland and Janzen (1974) hypothesized that mammalian herbivores might rely on gut bacteria to detoxify secondary compounds of novel plants during host shifts, and a similar bacterial role has been suggested for insects (Broderick et al., 2004; Dillon and Dillon, 2004; Janson et al., 2008). External microbial associates, including yeasts, also have great detoxifying potential for saprophagous species that must also contend with plant secondary chemistry. Because associations with symbiotic microbes are likely to be more evolutionarily labile than endogenous detoxification mechanisms, symbiosis might facilitate colonization of and adaptation to novel plant substrates.

The detoxification abilities of *Drosophila*-associated yeasts have been extensively demonstrated in the cactophilic *Drosophila. Diplodocus* and *Pichia* species found on *Stenocereus thurberi* cacti hydrolyze plant lipids that inhibit both larval fly and yeast growth (Starmer, 1982) while *Candida* and *Cryptococcus* species consume byproducts of cactus fermentation (2-propanol and acetone), which are toxic to *Drosophila mojavensis* (Starmer et al., 1986). Fungi associated with other insects degrade a wide variety of common plant secondary compounds, including tannins, terpenes, chlorinated hydrocarbons, and phenolics, among others (Dowd, 1992). The Hawaiian *Drosophila* utilize a chemically diverse collection of host plants and may similarly benefit from the detoxifying activity of yeasts.

Examples of detoxification by insect gut bacteria are limited, but several recent reports suggest this phenomenon may be more widespread than is currently appreciated. Terpene-degrading bacteria are associated with pine bark beetles (*Dendoctronus ponderosae*) that mine galleries in terpene-rich subcortical tissues of pines (Adams et al., 2013; Boone et al., 2013), and gypsy moths (*Lymantria dispar*) rely upon plant-derived bacteria to supplement endogenous detoxification of phenolic glycosides (Mason et al., 2014). These results suggest that environmentally acquired bacteria can be important contributors to insect fitness by directly detoxifying plant compounds, which may facilitate invasion of novel niches.

Plant-associated bacteria may also contribute to insect detoxification capacities via horizontal gene transfer from ingested bacteria to gut residents. Such a transfer has been described in humans, where the bacterium *Bacteroides plebeius* from Japanese populations apparently acquired genes from marine Bacteroidetes that degrade seaweed polysaccharides (Hehemann et al., 2010). Like human guts, insect guts are hotspots of horizontal gene transfer (reviewed in Dillon and Dillon, 2004). Conjugative plasmids are shared promiscuously within the guts of silkworm larvae (Watanabe et al., 1998), although gut conditions may not be conducive to natural transformation (Ray et al., 2007). Some pathways for plant secondary compound detoxification are encoded in small genomic regions that might facilitate their transfer, such as genes in the pyrrolidine pathway responsible for nicotine catabolism in plant-associated *Pseudomonas putida* (Tang et al., 2013). Whether gene transfer from environmental bacteria to *Drosophila* gut bacteria is common is not yet known.

## CONCLUDING REMARKS

Understanding the factors that generated and maintain the staggering diversity of the Hawaiian Drosophilidae has informed general hypotheses of how other organisms diversify. The diversity of these flies appears to be due to many different factors including geography, mating behaviors, and ecology. We propose that the interaction between the Hawaiian Drosophilidae, their host plants, and host-associated microbes is another important aspect driving the diversification of the Hawaiian Drosophilidae and/or maintaining this diversity. The role of microbial associates in nearly every aspect of drosophilid ecology—development, nutrition, host finding, and reproduction—presents many opportunities for those microbes to influence diversification. Experimental data from other *Drosophila* species suggest that bacteria, yeast and other fungi may allow host shifts and even trophic shifts, as well as instigate major changes in mating behaviors that subdivide populations in the Hawaiian Drosophilidae. Because yeasts are critical to host finding and substrate processing, Hawaiian *Drosophila* may not be radiating on host plants directly, but instead on fungal diversity. Additional studies are poised to expose the importance of these interactions.

## Conflict of Interest Statement

The authors declare that the research was conducted in the absence of any commercial or financial relationships that could be construed as a potential conflict of interest.
